# The impact of psychopathological subtypes on retention rate of patients with substance use disorder entering residential therapeutic community treatment

**DOI:** 10.1186/s12991-016-0119-x

**Published:** 2016-11-08

**Authors:** Angelo G. I. Maremmani, Pier Paolo Pani, Emanuela Trogu, Federica Vigna-Taglianti, Federica Mathis, Roberto Diecidue, Ursula Kirchmayer, Laura Amato, Joli Ghibaudi, Antonella Camposeragna, Alessio Saponaro, Marina Davoli, Fabrizio Faggiano, Icro Maremmani

**Affiliations:** 1 Vincent P. Dole Dual Diagnosis Unit, Department of Neurosciences, Santa Chiara University Hospital, University of Pisa, Via Roma, 67, 56100 Pisa, Italy; 2 Association for the Application of Neuroscientific Knowledge to Social Aims (AU-CNS), Pietrasanta, Lucca, Italy; 3 Social and Health Services, Cagliari Public Health Trust (ASL Cagliari), Cagliari, Italy; 4 Department of Psychiatry, Cagliari Public Health Trust (ASL Cagliari), Cagliari, Italy; 5 Piedmont Centre for Drug Addiction Epidemiology, ASLTO3, Grugliasco, Turin, Italy; 6 Department of Clinical and Biological Sciences, San Luigi Gonzaga University, Turin, Italy; 7 Department of Epidemiology, Latium Regional Health Service, Rome, Italy; 8 National Coordination Hospitality Communities (CNCA), Rome, Italy; 9 Regional Epidemiological Observatory, Emilia Romagna Regional Health Service, Bologna, Italy; 10 Department of Translational Medicine, Avogadro University, Novara, Italy; 11 G. De Lisio Institute of Behavioural Sciences, Pisa, Italy

**Keywords:** Addiction, Alcohol, Cocaine, Dropout, Heroin, Psychopathology, Retention, SCL-90, Therapeutic community treatment

## Abstract

**Background:**

A specific psychopathology of addiction has been proposed and described using the self-report symptom inventory (SCL-90), leading to a 5-factor aggregation of psychological/psychiatric symptoms: ‘worthlessness and being trapped’, ‘somatic symptoms’, ‘sensitivity-psychoticism’, ‘panic-anxiety’ and ‘violence-suicide’ in various populations of patients with heroin use disorder (HUD) and other substance use disorders (SUDs). These clusters of symptoms, according to studies that have highlighted the role of possible confounding factors (such as demographic and clinical characteristics, active heroin use, lifetime psychiatric problems and kind of treatment received by the patients), seem to constitute a trait rather than a state of the psychological structure of addiction. These five psychopathological dimensions defined on the basis of SCL-90 categories have also been shown to be correlated with the outcomes of a variety of agonist opioid treatments. The present study aims to test whether the 5-factor psychopathological model of addiction correlates with the outcome (retention rate) of patients with SUDs entering a therapeutic community (TC) treatment.

**Methods:**

2016 subjects with alcohol, heroin or cocaine dependence were assigned to one of the five clusters on the basis of the highest SCL-90 factor score shown. Retention in treatment was analysed by means of the survival analysis and Wilcoxon statistics for comparison between the survival curves. The associations between the psychopathological subtypes defined by SCL-90 categories and length of retention in treatment, after taking into account substance of abuse and other sociodemographic and clinical variables, were summarized using Cox regression.

**Results:**

Patients with cocaine use disorder (CUD) showed poorer outcomes than those with heroin dependence (HUD). Prominent symptoms of “worthlessness-being trapped” lead to a longer retention in treatment than in the case of the other four prominent psychopathological groups. At the multivariate level, age, detoxified status and total number of psychopathological symptoms proved to influence outcome negatively, especially in CUD. Somatic symptoms and violence-suicide symptoms turned out to correlate with dropout from residential treatment.

**Conclusions:**

The SCL-90 5-factor dimensions can be appropriately used as a prognostic tool for drug-dependent subjects entering a residential treatment.

## Background

The identification of a specific psychopathology of heroin use disorders (HUDs) is a major issue that has only been addressed recently [1, 2]. Even though addiction has often been labelled as a form of mental illness, there is no consensus so far about the core of this disease or the clinical covariates of addictive behaviours. Craving, in fact, which deserves to be considered one of the main features of addiction, was only added as a diagnostic criterion in the latest version of DSM [3]. The question of the existence of a specify psychopathology of addiction arises from the evidence of a high degree of association between the core symptoms of addiction and symptoms of other psychiatric diseases [4–6]. Moreover, further neurobiological and clinical considerations highlight the strong sharing of features between addiction per se and other psychopathological disorders, especially in the mood, anxiety and impulse/control domains, thus querying the classical model of psychiatric ‘comorbidity’ [7].

Initially, by applying an exploratory principal component factor analysis (PCA) to the 90 items in the SCL-90 checklist in a sample of 1055 heroin addicts entering agonist opioid treatment (AOT), a 5-factor solution was identified: the first factor reflected a depressive ‘worthlessness and being trapped’ dimension; the second factor picked out a “somatic symptoms” dimension; the third identified a ‘sensitivity-psychoticism’ dimension; the fourth a ‘panic-anxiety’ dimension; and the fifth a ‘violence-suicide’ dimension. [1]. The same methodology applied in a different sample of 1195 HUD subjects entering a therapeutic community (TC) Treatment led to the identification of the same five psychopathological dimensions [2]. Sociodemographic factors, clinical characteristics such as active heroin use, lifetime psychiatric problems, and kind of treatment received by the patient did not seem to substantially influence the five SCL-90 defined aggregation of symptoms [2, 8, 9].

Given the high susceptibility of patients suffering from substance dependence to leaving treatment programmes, retention has been historically regarded as a proxy for the effectiveness of interventions. Research on this topic has explored the potential predictors of retention in treatment by investigating sociodemographic profiles, clinical conditions and treatment-related factors. Given the high prevalence of psychiatric symptoms or overt psychiatric conditions in substance use disorders, the impact of their presence or severity has properly been considered to be one of the determinants of treatment retention [10–18].

The present study aims to test if the 5-factor solution psychopathological model of addiction correlates with outcome (retention rate) of SUD subjects entering a TC Treatment.

## Methods

### Design of the study

A prospective longitudinal approach was performed on the evaluation of therapeutic community treatments and outcomes (The VOECT) cohort study. The VOECT study was conducted in eight Italian regions in 2008–2009, recruiting a total of 2533 patients entering a TC treatment for substance use disorder [19]. For the present study, specific inclusion criteria were applied: (i) minimum age of 18, (ii) diagnosis of heroin, cocaine or alcohol substance use disorder (SUD) based on a clinical judgment, (iii) outcome data (dropout from TC treatment). Sociodemographic information and replies to SCL-90 questionnaires were collected at the baseline (at entry into treatment). These criteria lead to a definitive sample of 2016 subjects.

All subjects examined filled in an informed consensus document to enable them to participate in this study. The study was conducted in accordance with internationally accepted criteria and dispositions for ethical research.

### Sample

The sample consisted of 2016 SUD patients diagnosed according to a clinical judgement; 1693 (84.0%) of them were males and 323 (16.0%) females. At the time of the recruitment, the average age of the sample was 35.28 ± 8.6 years (minimum 18, maximum 74). Length of education was less than 8 years in 1600 (79.4%) patients. 1781 (88.3%) were single. 1598 (79.3%) were unemployed. 1471 (73.0%) subjects lived at home and 545 (27.0%) alone.

### Instruments

#### Self-report symptom inventory (SCL-90)

Developed by Derogatis and colleagues [20], the SCL90 consists of 90 items, each rated on a 5-point scale of distress. It is a self-report clinical rating scale oriented to the collection of symptomatic behaviours of psychiatric outpatients. Among heroin-dependent patients, the 90 items reflected the five primary symptom dimensions which are believed to underlie the large majority of symptom behaviours observed in this kind of patient: worthlessness-being trapped, somatic symptoms, sensitivity-psychoticism, panic-anxiety and violence-suicide [1].

The ‘worthlessness-being trapped’ dimension reflects a broad range of the symptoms typical of the clinical depressive syndrome. This dimension mirrors feelings of worthlessness and of being trapped or caught. The ‘somatic symptoms’ dimension reflects distress arising from perceptions of body dysfunctions. The ‘sensitivity-psychoticism’ dimension focuses on feelings of a full continuum of psychotic behaviours. The ‘panic-anxiety’ dimension subsumes a set of symptoms and experiences usually clinically associated with a high level of manifest anxiety. The ‘violence-suicide’ dimension is organized around three categories of hostile behaviour: thoughts, feelings, and actions; it also comprises thoughts of death and suicidal ideation.

In several previous studies, these five dimensions were empirically established and validated [2, 8, 9, 21]. SCL-90 was administered within 15 days after entry into the TC programme.

#### Other instruments

Information on the sociodemographic and clinical characteristics of the patients included in the study was collected through a research questionnaire administered at the time of entering TC.

### Data analysis

The sample was divided into three groups according to the primary substance of abuse (Alcohol, Heroin or Cocaine). We further classified patients according to which of the five SCL-90 dominant dimensions were found in each of them. SCL-90 factor scores were standardized into *z* scores in order to make scores comparable. Each subject was assigned to one of the five subtypes on the basis of the highest *z* scores achieved (named “prominent psychopathological dimensions”). This procedure gives the opportunity to classify subjects on the basis of the highest symptomatological cluster, thus overcoming the problem of identifying a cut-off point for the inclusion of patients in the clusters. The subtypes are clearly distinct, as shown by analysing the mean z-scores and 95% confidence interval (CI 95%) across the factors for each dominant group [1].

Retention in treatment was analysed by means of the survival analysis and Wilcoxon statistics for comparison between the survival curves. For the purpose of this analysis, the term ‘censored observations’ refers to patients who were still in treatment at the end of the study or were leaving treatment for reasons unrelated to the treatment itself (e.g. patients moving on other therapeutic communities, due to imprisonment for old crimes). We considered it to be a negative outcome (terminal event) when a patient abandoned the residential treatment or was expelled from the residential treatment. We compared the patients’ survival rates according to the primary substance of abuse and according to the psychopathological subtypes. The association between psychiatric subtypes and retention in treatment was summarized using Cox regression. In our model, we included sociodemographic and clinical variables that may act as confounding factors (age, gender, marital status, detoxification status, living conditions, primary substance of abuse, severity of psychopathological symptoms).

We used the statistical routines of SPSS, version 20.0.

## Results

2016 patients were observed for 1 month, 1724 for 2 months, 1451 for 3 months, 1207 for 4 months, 861 for 5 months, 692 for 6 months, 541 for 7 months, 439 for 8 months, 346 for 9 months, 269 for 10 months, 206 for 11 months, 161 for 12 months, 126 for 13 months, 86 for 14 months 47 for 15 months and 9 for 16 months. At the end of the study, the cumulative retention rate was 0.39.

### Retention rate according to the primary substance of abuse

**Fig. 1:**
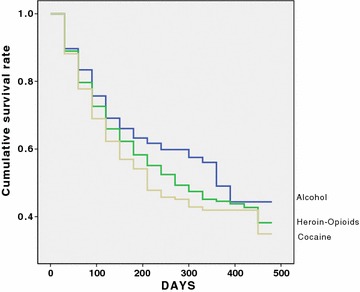
Retention rate according to the primary substance of abuse of 2016 SUD patients treated in a therapeutic community

The primary substance of abuse was alcohol in 401 (19.9%) of the part, heroin or other opioids in 1045 (51.8%) and cocaine in 570 (28.3%). Figure 1 shows retention rate according to the primary substance of abuse. Retention rates differed statistically between the three subgroups (Wilcoxon statistics = 6.59; *df* = 2; *p* = 0.037). In particular, 64.3% of the patients primarily using alcohol were censored, compared with 57.4% of the patients primarily using cocaine (Wilcoxon statistics = 6.54; *df* = 1; *p* = 0.011).

### Retention rate according to the prominent psychopathology at residential treatment entry

**Fig. 2:**
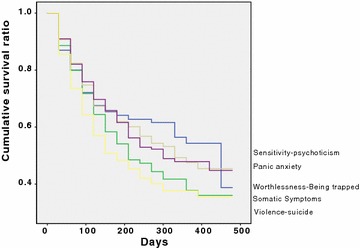
Retention rate according to the prominent psychopathology of 2016 SUD patients treated in a therapeutic community

The ‘worthlessness-being trapped’ dimension was prominent in 298 (14.8%) patients, ‘somatic symptoms’ in 456 (22.6%), ‘sensitivity-psychoticism’ in 406 (20.1%); ‘panic-anxiety’ in 518 (25.7%) and ‘violence-suicide’ in 338 (16.8%). Figure 2 shows the retention rate according to the psychopathological subtypes. Retention rates differed statistically between the five subgroups (Wilcoxon statistics = 17.19; *df* = 4; *p* = 0.002). In particular, patients with prominent ‘violence-suicide’ symptomatology showed a poorer retention rate (52.7% of those entering a treatment) than patients with prominent ‘worthlessness-being trapped’ (65.4% of entrants; Wilcoxon statistics = 6.02; *df* = 1; *p* = 0.014), with prominent ‘somatic symptoms’ (57.0% of entrants; Wilcoxon statistics = 4.35; *df* = 1; *p* = 0.037), with prominent ‘sensitivity psychoticism’ (63.8% censored; Wilcoxon statistics = 11.60; *df* = 1; *p* = 0.001) and with prominent ‘panic anxiety’ symptomatology (63.3% of entrants; Wilcoxon statistics = 11.60; *df* = 1; *p* = 0.000),

### Correlation between primary substance of abuse together with prominent psychopathology and residential treatment outcome

Table 1 shows the correlation between primary substance of abuse together with prominent psychopathology and residential treatment outcome corrected by demographic and clinical characteristics. Only age correlated negatively with dropping out of residential treatment. Oldest patients tended to remain in treatment longer than the youngest ones. Cocaine use disorder patients remained in treatment for a shorter time than heroin use disorder ones. Not having been detoxified at residential treatment entry and the presence of psychopathological symptoms were the two factors that influenced the outcome negatively. The prominent ‘somatic symptoms’ and ‘violence-suicide’ symptomatology at treatment entry correlated positively with dropout from the therapeutic community.

**Table 1:** Correlations between residential treatment negative outcomes and associated covariates

Variables	*B*	Exp(*B*)	95% CI	Sig
Age	−0.02	0.98	0.98–0.99	0.001
Gender (female)	−0.06	0.94	0.77–1.15	0.566
Civil status (with partner)	−0.05	0.96	0.75–1.21	0.707
Education (lasting <8 years)	0.02	1.02	0.85–1.22	0.835
Living with parents	−0.05	0.95	0.81–1.12	0.548
Entering therapeutic communities without having been detoxified	0.58	1.79	1.55–2.07	0.000
Total SCL90 at treatment entry	0.002	1.002	1.001–1.004	0.000
SCL90 typology^a^				0.015
Prominent somatic symptoms	0.27	1.31	1.03–1.67	0.028
Prominent sensitivity-psychoticism symptoms	0.08	1.09	0.84–1.40	0.520
Prominent panic-anxiety symptoms	0.16	1.17	0.92–1.49	0.207
Prominent violence-suicide symptoms	0.38	1.46	1.14–1.87	0.003
Primary substance of abuse^b^				0.036
Alcohol	0.03	1.04	0.84–1.27	0.742
Cocaine	0.21	1.23	1.05–1.45	0.011
Statistics: Chi square 131.21, *df* 13, *p* = 000				

^a^Considering prominent ‘worthlessness-being trapped’ as reference group

^b^Considering heroin as the reference primary substance of abuse

## Discussion

The ‘worthlessness-being trapped’ psychopathological domain leads to a longer retention in treatment than the other four prominent psychopathological groups. On the other hand, the ‘somatic symptoms’ and ‘violence-suicide symptoms’ groups correlate with dropout from residential treatment. When considering various different drugs of dependence, cocaine use disorder (CUD) patients show outcomes worse than HUD individuals. At a multivariate level, age, detoxified status and total number of psychopathological symptoms influence outcomes negatively, especially in the case of CUD.

The severity of psychopathology appears to have a crucial impact on retention rate in treatment progression. In line with this, the presence of psychiatric comorbidity has been shown to have a general negative prognostic significance when opioid maintenance treatment programmes were studied [11, 22–24]. Wide discrepancies in results have, however, been found [13–16, 18, 25] with several studies showing no influence on retention in treatment [14–18, 25, 26], whereas other studies reported a substantial difference in retention, whether based on the presence of a mental disorder [27] or in considering specifically psychopathological traits, such as those located on DSM Axis II [13, 28]. In addition, the impact of psychiatric comorbidity has been studied in TCs, whereas the highest dropout frequency has been observed in patients with the highest severity of psychiatric problems [29, 30].

Moreover, the correlation between psychopathology severity and retention in treatment increases substantially when looking at specific psychopathological dimensions. While the severity of ‘sensitivity-psychoticism’ and ‘panic-anxiety’ symptoms did not turn out to interfere with retention, patients belonging to the ‘somatic symptoms’ and ‘violence-suicide’ dimensions showed a 1.31- and 1.46-fold probability, respectively, of leaving their treatment compared with those belonging to the ‘worthlessness-being trapped’ dimension. Physical complaints may be a part of the psychopathological structure of addiction, but may also be consistent with the somatic consequences of the use of substances, partly as a direct effect of their use on specific organs and functions, other than intoxication and withdrawal. Moreover, physical complaints must be partly attributed to the risk-taking style of lives associated with addiction. Lastly, “somatization” has been viewed as a component of the addictive personality, and has been correlated with probable dropout from therapeutic programmes [31]. It is, however, easy to understand that these complaints tend to act in the same way as a stressful condition that induces patients to leave their treatment. On the other hand, the high probability of leaving TCs that is found in patients with ‘violence-suicide’ symptomatology may be explained in the light of the psychological and behavioural features of this very psychiatric dimension. If we now go through the SCL-90 items, besides suicidal thoughts or longings for death, patients belonging to this dimension have difficulty in controlling their impulsiveness and rage. In cases with this psychological background, it should be easy to understand that these patients are likely to show intolerance towards peer-based social interactions, to a clear system of rules and regulations, and to the approach of ensuring that each patient has a long stay in the community, all of which supply basic criteria underlying a therapeutic community [32]. Moreover, it is critical to note that TC programmes are often distinguished by their tendency to avoid a medical approach or any specifically psychiatric treatments. Consistently with this explanation, ‘violence-suicide’ dominant dimensions turned out to be associated with a preference for OAT when compared with a TC programme [2], due to the positive effects of therapeutic opioids on patients’ psychopathology [33].

Being older is associated with longer retention in treatment. Easy dropout from treatments designed to combat the substance dependence of young people was a feature noted in previous studies carried out with large samples of participants, although other studies failed to identify a correlation between age and length of stay in TCs [34]. Data from studies that looked specifically at dropout from residential TCs are sparse and controversial: a majority of studies fail to show any correlation between age and retention [35–37]; there has, however, been at least one study that shows such a correlation [38]. The higher level of impulsivity and risk-taking behaviour shown by adolescents and correlated with the process of development of brain structures has been hypothesized as the explanation for the high likelihood of leaving treatment [39]. Being compliant with TC treatment is surely a sign of awareness of illness, and results from this study suggest that older addicts show a better level of insight than younger ones, in line with previous observations providing evidence that the presence of insight correlates with the progression of the toxicomanic process [40].

When different drugs of addiction are compared, CUD patients show the highest dropout levels from TC programmes. This result may depend on the effects exerted by cocaine on mood and psychological performance. It must be considered that sample size (about 2000 participants) could partly explain the differences that emerge from our findings. In any case, the high frequency of such dropouts has even been confirmed by Cox regression, which documented the confounding effects of psychopathology. Considering the kind of substance abused, some studies have shown an association of cocaine use with dropout [41–45], but this has not been confirmed in studies carried out in therapeutic communities [35, 38, 46].

The strong correlation between intoxication status and dropout from TC programmes can hardly be considered surprising. The finding that the probability of dropout in people who had not been detoxified was almost double (exp. 1.79) that of detoxified patients can easily be understood considering the extreme susceptibility of patients with addiction problems to cues and conditions related to drug use, and the primary role played by withdrawal in craving and relapse. On these bases, it is easy to understand how a patient complaining about physical and psychological symptoms may choose to dropout of residential treatment. This may be observed especially in abstinence-oriented TC programmes, which appear to lead to undervaluation of the impact of withdrawal on willingness to stay in treatment.

## Limitations

As to limitations, it must be considered that some of the important determinants of retention in treatment, including those that may be associated with psychopathological severity, were left out of consideration in this study. First, only heroin, cocaine and alcohol were considered as the primary substance of abuse, while the possible secondary use of these substances or the use of other substances (including cannabis, nicotine and hallucinogens) as possible determinants of dropout was not discussed. Second, the presence of specific formal psychiatric diagnoses was not recorded: the availability of formal psychiatric diagnoses, besides giving information on the association between specific mental disorders and dropout, would also have made it possible to look at potential correlations between psychiatric disorders and psychopathological dimensions. Third, interventions carried out in TCs in the form of delivering psychological and psychiatric care, the availability of pharmacological interventions and the presence of psychosocial services were not collected; all these are factors that may act as confounders of the relationship between psychopathology and retention in treatment.

Other limits to the validity of the five SCL-90-based psychopathological dimensions solution have been discussed in previous studies on the SCL-90-defined structure of the psychopathology of opioid addiction [1] and on the same population [2, 8, 9]. In these studies, the neglected factor has been the lack of any observer-related ‘objective’ evaluation, as SCL-90 is a self-administered instrument that may be affected by the voluntary or involuntary hiding of some symptoms.

Finally, a further limitation is that in this analysis the SCL-90 questionnaire was administered at treatment entry only, and hence, results can only be considered representative of subjects with addiction at that initial moment. Some symptoms may vary at different stages of the disease so that they may prove to be under- or overweighed in our sample.

## Conclusions

Length of retention in treatment of patients entering TC treatment is significantly lower for those who have a more severe psychopathology. Moreover, patients with prominent ‘violence-suicide’ and ‘somatic’ symptoms may leave the treatment earlier than those allocated to the other three psychopathological dimensions resulting from the application of PCA to the SCL-90 responses (i.e. ‘worthlessness-being trapped’, ‘sensitivity-psychoticism’ and ‘panic-anxiety’). The SCL-90 five-factorial structure of the psychopathology of substance dependence could turn out to be a useful tool when applied as a prognostic factor, together with age, detoxification status and kind of substance of abuse, all of which have been shown to influence retention in treatment at multivariate level.

## Abbreviations

AOT: agonist opioid treatment
CUD: cocaine use disorder
DSM: diagnostic statistic manual
PCA: principal component analysis
SCL-90: symptomatological check list-90
SUD: substance use disorder
TC: therapeutic community
VOECT: evaluation of therapeutic community treatments and outcomes
